# Role of the *ABCG8* 19H risk allele in cholesterol absorption and gallstone disease

**DOI:** 10.1186/1471-230X-13-30

**Published:** 2013-02-13

**Authors:** Olga Renner, Dieter Lütjohann, Dominique Richter, André Strohmeyer, Silke Schimmel, Oliver Müller, Eduard F Stange, Simone Harsch

**Affiliations:** 1 Dr. Margarete Fischer-Bosch Institute of Clinical Pharmacology and University of Tuebingen, 70376, Stuttgart, Germany; 2 Institute for Clinical Chemistry and Clinical Pharmacology, Laboratory for Special Lipid Diagnostics, University of Bonn, Germany; 3 Department of Gastroenterology, Robert Bosch Hospital, 70376, Stuttgart, Germany

## Abstract

**Background:**

Gallstone disease is associated with p.D19H of *ABCG8* as well as alterations of cholesterol and bile acid metabolism. However, molecular mechanisms have not been fully elucidated. It is important to understand the link between the sterol transporters ABCG5/8 and NPC1L1 and intestinal cholesterol absorption as well as *de novo* synthesis in gallstone patients stratified according to 19H risk allele. Moreover, the functional importance of the 19H variant on intestinal ABCG8 feature remains to be clarified.

**Methods:**

Measurements of serum surrogate markers of cholesterol absorption (plant sterols: sitosterol, campesterol) and synthesis (cholesterol precursor: lathosterol) were carried out by gas chromatography/mass spectrometry (GC/MS). For expression studies, total RNA was isolated from 168 ileal biopsies of study participants with (34) and without gallstone disease (134). Messenger RNA was measured by LightCycler real-time PCR. Genomic DNA was obtained from blood leukocytes. Genotype frequencies of p.D19H were established using MALDI-TOF mass spectrometry.

**Results:**

Compared to controls, cholesterol absorption but not synthesis in gallstone carriers was diminished by about 21% based on low serum sitosterol (*P* = 0.0269) and campesterol (*P* = 0.0231) to cholesterol ratios. D19H was found to be significantly associated with gallstones (odds ratio [OR] = 2.9, *P* = 0.0220, 95% confidence interval [CI]:1.22-6.89), particularly in the overweight cohort (OR = 3.2, *P* = 0.0430, 95% CI:1.07-9.26). Cholesterol absorption was about 24% lower in individuals carrying p.D19H compared to wild type (*P*_sitosterol_ = 0.0080, *P*_campesterol_ = 0.0206). Moreover, irrespective of phenotype, carriers of p.D19H displayed a significant lower absorption than carriers of the major allele. The most pronounced effect on cholesterol absorption ratio was observed for serum campesterol levels (wild type controls to mutated controls 28%, *P* = 0.0347 and wild type controls to gallstone carriers with 19H allele 37%, *P* = 0.0030). Notably, ABCG5/8 and NPC1L1 expression was similar in gallstone carriers and controls regardless of p.D19H presence.

**Conclusions:**

Both gallstone disease and p.D19H of *ABCG8* are associated with diminished cholesterol absorption. However, p.D19H is not responsible for the differences in small intestinal sterol transporter expression.

## Background

Gallstone disease is a frequent health problem affecting 10-20% of the Western population [1]. An imbalance between cholesterol, bile acids and phospholipids is a key factor for cholesterol gallstone formation [2] but the metabolic cause of this disturbance is still unclear. Gallstone carriers and controls exhibit basic differences in cholesterol and bile acid homeostasis [3,4]. In the presence of normal dietary cholesterol its absorption tended to be diminished in gallstone carriers whereas synthesis was induced [3]. However, the contribution of newly synthesized to biliary cholesterol in animals and man is small [5-8] and the origin of excess biliary cholesterol remains uncertain. Moreover, gallstone carriers respond to an increased cholesterol load with an elevated biliary cholesterol secretion and exhibit a diminished hepatic *de novo* cholesterol synthesis [3]. Furthermore, the regulation of cholesterol synthesis in overweight persons, prone to develop gallstones, differs from the lean individuals and is feedback inhibited after the application of long-term high cholesterol diet [9]. Gallstone carriers also exhibit a reduction in the primary bile acid pool size, an increase of the biliary fraction of secondary bile acids, reduced gallbladder motility as well as a prolonged intestinal transit time [10,11]. The mechanism behind the low primary bile acids in bile may be a diminished expression of bile acid transporters in the terminal ileum [12,13]. The ultimate consequence is cholesterol supersaturation in the gallbladder bile, leading to crystal precipitation of free cholesterol and gallstone formation [2,3,10,14].

Responsible for cholesterol transport in the human gut are the heterodimeric ATP-binding cassette transporter (ABCG5/8) and Niemann-PickC1-Like 1 Protein (NPC1L1). Following incorporation into micelles, dietary as well as biliary cholesterol is available for uptake from the intestinal lumen through the importer NPC1L1 [15]. This protein is localized at the apical membrane of the enterocytes [16] and contributes to intestinal cholesterol homeostasis [15-18]. The heterodimeric sterol transporter ABCG5/8 determines hepatobiliary cholesterol secretion and cholesterol efflux out of the enterocytes back into the intestinal lumen, thereby promoting net cholesterol removal from the body [19-21]. Moreover, the intestinal cholesterol transporters are under the transcriptional control of liver X receptors (LRXα/β), sterol regulatory element-binding protein (SREBP2), hepatocyte nuclear factors (HNF1α/4α) and peroxisome proliferator-activated receptor (PPARδ) [18,22-25].

Human and murine studies support a strong genetic background of gallstone risk [26-28]. Most prominently associated with gallstone disease is the D19H polymorphism of the *ABCG8* gene [29]. In healthy individuals, the D19H polymorphism was associated with low cholesterol absorption [30]. Similarly, Gylling described low serum total cholesterol and cholesterol absorption in the cohort of mildly to moderately hypercholesterolemic subjects with the 19H allele [31]. Recently, the study of Krawczyk showed that cholesterol absorption was significantly lower and *de novo* synthesis higher in gallstone carriers [4] but this was independent of the D19H polymorphism.

The present study therefore addressed the following questions: i) are both, low cholesterol absorption and increased *de novo* cholesterol synthesis indeed common in gallstone carriers, ii) is the expression of intestinal cholesterol transporters and their transcription factors different in gallstone carriers, iii) does the D19H polymorphism of the *ABCG8* gene affect any of these parameters?

## Methods

### Ethics statement

The local ethics committee (ethics committee of the University Hospital of Tuebingen and University Tuebingen) approved this study and all subjects gave written informed consent prior to participation.

### Subjects

Study subjects were recruited from the contingent of individuals invited to undergo routine colonoscopy according to the recommendations of German Health Organisation and for cancer prevention. Individuals, who agreed the participation in the study, were personally interviewed and seen by the physicians regarding their health conditions. Subjects included in this study had *a)* normal serum lipid values and no history of taking lipid-lowering drugs or drugs interfering with bile acid uptake, *b)* no known medical conditions affecting lipid metabolism, *c)* normal liver function and no signs of haemolysis or other conditions associated with pigment stones, *d)* no intestinal surgery and *e)* no impaired nutritional status. None of the gallstone carriers or controls had symptomatic gallstone disease, abnormal liver function, elevated serum lipids or inflammation in the ileum. Biopsies and blood samples were collected from a total of 168 individuals in the Robert Bosch Hospital in Stuttgart. Blood samples (3–5 mL) were used for cholesterol and phytosterol measurements as well as for genotyping investigations. Up to eight ileal biopsy specimens were taken from each participant (for this investigation two separate samples, each about 8–10 mg, were used). 134 subjects were healthy controls and 34 individuals had gallstones. The presence or absence of gallstones was confirmed by ultrasound. Serum triglycerides and cholesterol levels were analysed by standard clinical tests.

### Measurements of sterol synthesis and absorption

Measurements of the non-cholesterol sterols lathosterol, campesterol and sitosterol from serum samples were carried out by GC-MS as reported previously [32]. Cholesterol absorption was given as ratio of sitosterol or campesterol to total cholesterol and cholesterol synthesis was calculated as the ratio of lathosterol to cholesterol [33].

### Real-time quantitative reverse transcription – polymerase chain reaction (RT-PCR)

Total RNA was isolated from whole biopsy specimen using TRIzol extraction procedure (Invitrogen) according to the manufacturer’s protocol. The integrity, quality and quantity of RNA were analysed by gel electrophoresis and absorption measurement. Four hundred ng of total RNA was reverse transcribed using the (AMV)-reverse transcriptase system (Promega) and random hexamers. RT-PCR was performed with LightCycler sequence detection system (Roche Diagnostics) as reported previously [12,13]. Primer sequences used for amplification of human ABCG5, ABCG8, NPC1L1, LXRα, LXRβ, HNF1α, HNF4α, PPARδ and SREBP2 are listed in Table 1. Target specific PCR conditions were: 95°C for 5 min, 40 cycles at 95°C for 10 s, followed by 60°C for 5 s, then 72°C for 7 s. For the quantification of NPC1L1 a PCR of 50 cycles was necessary. At the end of the PCR, a dissociation curve was performed. The quantity for any given transcript was calculated using the second derivative maximum method according to manufacturer’s instructions. Gene-specific plasmids served as control templates with specific oligonucleotide primer pairs. All measurements were carried out in duplicate.

**Table 1:** Primer sequences used for QRT-PCR

**Fragment**	**Primer**	**Sequence**	**Product size**
Exon 9/10	ABCG5 F	5’ – CGCGTAGGTCTCCTTTACCA – 3’	152
	ABCG5 R	5’ – AGTGCATAGGCCAGCATCAT – 3’	
Exon 10/11	ABCG8 F	5’ – GTGGCTGGTGGTCTTCTGTT – 3’	84
	ABCG8 R	5’ – CTGAAGAAGGAGGCCATGTG – 3’	
Exon 17/18	NPC1L1 F	5’ – GTGGGGCATCAGTTACAATG – 3’	166
	NPC1L1 R	5’ – AAACACCGCACTTCCCATAG – 3’	
Exon 4/5	LXRα F	5’ – TCAGGCGGATCTGTTCTTCT – 3’	213
	LXRα R	5’ – TCAGGCGGATCTGTTCTTCT – 3’	
Exon 7/8	LXRβ F	5’ – TAAGCAAGTGCCTGGTTTCC – 3’	200
	LXRβ R	5’ – ACTCGAAGATGGGGTTGATG – 3’	
Exon 8/10	HNF1α F	5’ – CACGCCCACCAAGCAGGTCT – 3’	165
	HNF1α R	5’ – CCAGGCTGCTGGAGGACACTG – 3’	
Exon 9/10	HNF4α F	5’ – ATGCTTCCGGGCTGGC – 3’	235
	HNF4α R	5’ – TTCGAGTGCTGATCCG – 3’	
Exon 4/5	SREB2 F	5’ – CAGCCTCAGATCATCAAGACA – 3’	202
	SREB2 R	5’ – CTTTCTCTTGCCCCATCATTA – 3’	
Exon 4/5	PPARδ F	5’ – ATGGAGCAGCCACAGGAGGAAGCC – 3’	200
	PPARδ R	5’ – GCATGAGGCCCCGTCACAGC – 3’	

### Plasmid construct

Plasmids were constructed with the TOPO TA Cloning Kit (Invitrogen) according to manufacturer’s recommendations. Four μL PCR product were subcloned into the EcoRI sites of a 2.1 TOPO TA cloning vector. Transformation was performed with two μl of the ligation onset to TOP 10 cells (E. coli) and spread to agar plates appended with X-Gal and ampicillin. The sample was incubated overnight at 37°C. White colonies were picked and purified/isolated with the Miniprep kit (Qiagen). The positive clones were verified by DNA sequencing.

### Western blot analysis

For protein analysis a separate whole ileal mucosal biopsy was required. Total protein was isolated using TRIzol Reagent (Invitrogen). Protein concentration was determined applying the Bradford assay (Bio-Rad). Samples were heated at 95°C for 5 min. A total of six μg for ABCG8 and 15 μg for ABCG5/NPC1L1 protein were loaded and then electrophoresed (90 min, 100 V) on self-cast SDS-PAGE gels (ABCG5/8 10%, NPC1L1 8%) and blotted on nitrocellulose (0.45 μm, Whatman). After blotting, membranes were blocked for 1 h with 5% non-fat dry-milk (NFDM) in Tris-buffered saline containing 0.1% Tween 20 (TBST). Hereafter, membranes were incubated overnight with primary antibodies (ABGC5: ab45279/Abcam diluted 1:1,000 in 3% NFDM/TBST and 1% of Roti®-Block reagent (Roth); ABCG8: NB400-110/Novus Biologicals diluted 1:3,000 in 5% NFDM/TBST and NPC1L1:EPR5717/ab124801/Abcam diluted 1:200 in 3% NFDM/TBST). After being washed five times for 5 min blots were probed with the secondary antibody, peroxidise-conjugated goat anti-rabbit IgG (1:10,000, Dianova) and exposed to a chemiluminescent reagent (SuperSignal*T* West Dura; Pierce). The immunoreactive band was obtained at the predicted size of 75 kDa representing ABCG5 or ABCG8 and 145 kDa representing the NPC1L1 monomer. Bands were photographed and immunoquantitation was accomplished by densitometric analysis using the software AIDA (Raytest). To account for variability in the amounts of enterocytes in biopsy specimens, villin contents of all samples were determined. Therefore membranes were incubated with a primary antibody against human villin (1:2,000, Chemicon International), followed by incubation with the secondary peroxidise-conjugated anti-mouse IgG antibody (1:1,500, Oncogene). Protein staining was obtained at the predicted size of 95 kDa. All measurements were carried out in duplicate.

### Isolation of DNA

Genomic DNA was extracted from peripheral blood leukocytes by standard methods using the Qiagen system (QIAamp DNA blood kit/QIAGEN DNA Blood Midi Prep) according to manufacturer’s recommendations.

### Genotyping

The D19H SNP analysis of *ABCG8* was performed with blood DNA using matrix-assisted laser desorption/ionization time-of-flight mass spectrometry (MALDI-TOF MS) of allele specific primer extension products as reported previously [34], applying the following primer sequences:

1st-primer: ACGTTGGATGAGGAGAGAGGGCTGCCGAAA,

2nd-primer: ACGTTGGATGACTTCCCATTGCTCACTCAC and

extension primer: TGCTCACTCACCGAGGTATG.

For quality control no-template controls in all plates and repeated analysis of 10% of randomly selected samples were performed.

### Statistics

For statistical analysis GraphPad Prism version 5 was used (GraphPad Software, San Diego, Ca, USA). Clinical characteristics of study participants and all data are presented as means ± standard error of the mean (SEM). Differences between groups (anthropometric and metabolic characteristics (age, body mass index (BMI), total cholesterol and total triglycerides), cholesterol absorption and synthesis ratios and all expression data) were investigated using the two-tailed Mann–Whitney *U*-test. All correlations (of expression values as well as of cholesterol absorption and synthesis ratios) between variables were analysed with Spearman’s correlation rank test. Observed and expected genotype frequencies within the study population were compared by means of Hardy–Weinberg equilibrium calculations [35]. Data were considered in Hardy–Weinberg equilibrium when *P-*values were >0.05. Statistical analysis of genotype frequency differences between gallstone carriers and controls was done using two-sided Fisher’s exact test as appropriate. Odds ratios (ORs) together with a 95% confidence interval (CI) are given as risk measures for the development of gallstones. All statistical tests were two-tailed and a *P-*value of <0.05 was considered as statistically significant.

## Results

### Characteristics of the study cohort

Several relevant anthropometric and metabolic characteristics (age, serum cholesterol and triglyceride levels) of the participants did not differ significantly and are summarized in Table 2. Therefore, the total study cohort was stratified in two weight groups. The calculation of anthropometric data reveal a mean age of 57 years in the control group and 61 years in the gallstone carrier group. As weight also depends on age [36-40], in the present study the normal weight group was defined as BMI ≤ 25.4, individuals with BMI > 25.4 were regarded as overweight. Since the percentage of overweight subjects was higher among gallstone carriers (68%) than among control individuals (44%), the mean body mass index was slightly higher in the gallstone group (BMI = 26.6) compared to controls (BMI = 25.2) (*P* = 0.0378).

**Table 2:** Anthropometric and metabolic characteristics of the Stuttgart study cohort

	**Total**		**Normal weight**		**Overweight**	
**Parameter**	**Controls**	**Gallstone carriers**	**Controls**	**Gallstone carriers**	**Controls**	**Gallstone carriers**
Number	134	34	75	11	59	23
Age (years)	57 ± 1.1	61 ± 1.9	55 ± 1.5	63 ± 3.6	59 ± 1.4	60 ± 2.3
BMI (kg/m^2^)	**25.2 ± 0.3**	**26.6 ± 0.6**^**α**^	22.7 ± 0.3	23.3 ± 0.6	28.4 ± 0.4	28.2 ± 0.6
Total cholesterol (mg/dL)	214 ± 3.7	207 ± 6.2	211 ± 4.7	214 ± 6.3	217 ± 6.0	203 ± 8.8
Total triglycerides (mg/dL)	121 ± 6.1	126 ± 7.8	101 ± 6.8	125 ± 14.2	143 ± 9.9	127 ± 9.5
Lathosterol:Cholesterol (μg/mg)	1.15 ± 0.06	1.33 ± 0.15^**η**^	0.99 ± 0.07	1.22 ± 0.26	1.33 ± 0.09^**δ**^	1.39 ± 0.18
Sitosterol:Cholesterol (μg/mg)	**1.23 ± 0.06**	**0.97 ± 0.08**^**β**^	1.36 ± 0.09	1.11 ± 0.19	1.06 ± 0.07^**ε**^	0.90 ± 0.06
Campesterol:Cholesterol (μg/mg)	**1.47 ± 0.07**	**1.16 ± 0.11**^**γ**^	1.67 ± 0.12	1.23 ± 0.24	1.24 ± 0.08^**ζ**^	1.12 ± 0.11

*BMI* = body mass index.

Values are given as means ± SEM (standard error of the mean).

Significance between the controls and gallstone carriers was analysed with Mann–Whitney *U*-test (nonparametric, two-tailed).

Normal weight subgroup is defined as BMI ≤ 25.4, overweight subgroup as BMI > 25.4.

*P*-values <0.05 were considered as statistical significant.

Controls vs. gallstone carriers: ^**α**^*P* = 0.0378, ^**β**^*P* = 0.0269 (21%), ^**γ**^*P* = 0.0231 (21%), ^**η**^ (16%). No adjustment in the total group.

Normal weight persons vs. overweight individuals: ^**δ**^*P* = 0.0028 after adjustment (factor 6) *P* = 0.0168, ^**ε**^*P* = 0.0044 after adjustment *P* = 0.0264, ^**ζ**^*P* = 0.0065 after adjustment *P* = 0.039.

### Cholesterol absorption and synthesis in gallstone disease

Cholesterol absorption based on the serum sitosterol/cholesterol ratio correlated well with the campesterol/cholesterol ratio (ρ = 0.90, *P* < 0.0001, Spearman’s rank test). Interestingly, in the total cohort including both weight groups, cholesterol absorption was significantly lower by about 21% in gallstone carriers compared to controls (sitosterol *P* = 0.0269, campesterol *P* = 0.0231) (Table 2). The correlation between hepatic cholesterol synthesis and intestinal absorption was inverse and statistically significant (sitosterol/lathosterol ρ = −0.43, *P* < 0.0001; campesterol/lathosterol ρ = −0.38, *P* < 0.0001, Spearman’s rank test). In principle, these results were similar in female and male subpopulations when analysed separately, as shown in Additional file 1: Table S1C and D.

Overweight controls exhibited a higher synthesis (+33% *P* = 0.0028) and lower absorption (sitosterol −22% *P* = 0.0044; campesterol −26% *P* = 0.0065) rate than normal weight persons. Remarkably, in the subgroups the rate of cholesterol synthesis tended to be higher (4-22%, ns) and of cholesterol absorption lower (on average ~17%, ns) in gallstone individuals than in controls. However, none of these differences between gallstone carriers and controls were statistically significant.

### Ileal expression of sterol transporters and their relevant transcription factors in gallstone disease

**Figure 1:**
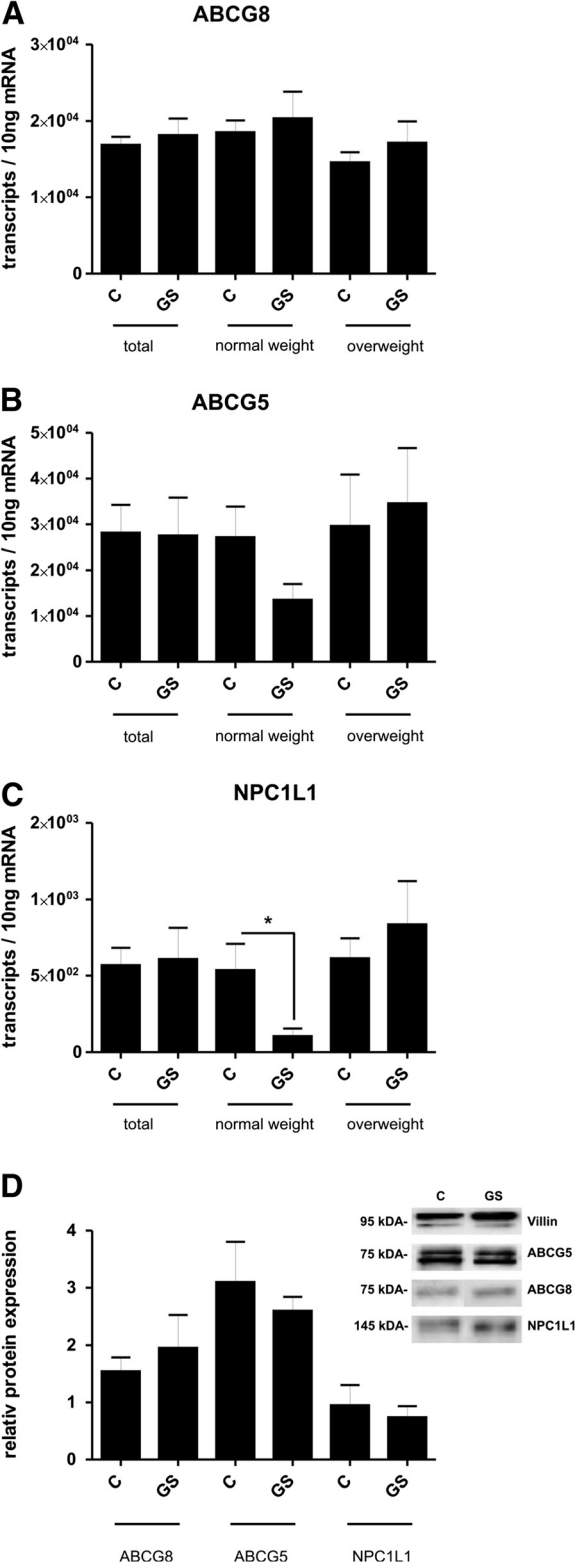
**Expression of cholesterol transporters in female gallstone carriers and healthy controls.** The expression analysis was performed in human ileal mucosal biopsies. Values are calculated as means ± SEM (standard error of the mean). Significance between the controls and gallstone carriers was analysed with Mann–Whitney *U*-test (nonparametric, two-tailed). Normal weight subgroup is defined as BMI ≤ 25.4, overweight subgroup as BMI > 25.4. *P*-values <0.05 were considered as statistically significant. (**A-C**) Quantification of mRNA expression is given as transcript numbers. ABCG5/ABCG8 = ATP-binding cassette transporter, NPC1L1 = Niemann-Pick C1-Like 1 protein *P = 0.0417, C = control subject, GS = gallstone carrier. Total: C = 98, GS = 30; normal weight: C = 57, GS = 10; overweight: C = 41, GS = 20. (**D**) Representative Western blot images of ABCG8, ABCG5 and NPC1L1 in ileal mucosa of gallstone carriers and controls. Protein content was determined by densitometric analysis. The data were normalized to villin, an epithelial marker protein. ABCG8: C = 70, GS = 20; ABCG5: C = 7, GS = 7; NPC1L1: C = 7, GS = 7.

Next, the ileal expression patterns of the three relevant sterol transporters ABCG8, ABCG5 and NPC1L1 were analysed (Figure 1). Regarding sterol exporter ABCG8 and ABCG5, no significant changes in expression were apparent when stratified according to gallstones (Figure 1A and B), also not within the different weight groups. In comparison with the exporters detailed above, ileal expression of the NPC1L1 importer was rather low (Figure 1C). Only normal weight stone-carriers exhibited a reduced expression by 80% compared to respective controls (*P* = 0.0417), whereas all other groups showed no relevant and significant expression differences. As shown in Figure 1D, comparable expression patterns could be stated on protein levels. The correlation analyses by Spearman’s rank test between transporters showed the following moderate to small correlation coefficients: ABCG5/ABCG8 ρ = 0.50, *P* < 0.0001; ABCG5/NPC1L1 ρ = 0.44, *P* < 0.0001 and ABCG8/NPC1L1 ρ = 0.12, *P* = 0.1812.

Furthermore, it was investigated whether gallstone disease is associated with alterations in the major regulatory transcription factors of the sterol transporters as summarized in Table 3. Only for HNF1α an increased transcript expression was observed in overweight gallstone carriers compared to relevant controls (*P* = 0.0470). None of the other regulatory factors exhibited any significant expression difference between controls and gallstone carriers in general, also not in the different weight groups. Finally, no clear expression correlation of the transcription factors to their respective transporters could be stated (data not shown).

**Table 3:** Mean values of mRNA transcript expression in ileal mucosa of important transcription factors in gallstone carriers and healthy controls

	**Transcripts/10 ng mRNA**					
	**Total**		**Normal weight**		**Overweight**	
**Group**	**Controls**	**Gallstone carriers**	**Controls**	**Gallstone carriers**	**Controls**	**Gallstone carriers**
**Target (Mean ± SEM)**	**(n = 95)**	**(n = 29)**	**(n = 55)**	**(n = 9)**	**(n = 40)**	**(n = 20)**
LXRα	70261 ± 4308	70004 ± 6774	75035 ± 6026	63318 ± 12203	63853 ± 5964	73544 ± 8245
LXRβ	36208 ± 2570	37337 ± 5445	39285 ± 3445	30067 ± 6295	32361 ± 3821	40781 ± 7426
HNF1α	5282 ± 362	7037 ± 864	5555 ± 488	5814 ± 953	**4944 ± 542**	**7616 ± 1183**^**a**^
HNF4α	48814 ± 3407	53086 ± 10701	51632 ± 4561	45244 ± 9990	44939 ± 5115	56615 ± 14955
PPARδ	7326 ± 574	7985 ± 999	7540 ± 806	6821 ± 1468	7060 ± 817	8537 ± 1304
SREBP2	29807 ± 2223	30681 ± 3864	31373 ± 3068	30677 ± 5749	27928 ± 3236	30683 ± 5145

Values are given as means ± SEM (standard error of the mean). Significance between the controls and gallstone carriers was analysed with Mann–Whitney *U* test (nonparametric, two-tailed). *P*-values <0.05 were considered as statistical significant.

*LXR* = liver X receptors, *HNF* = hepatocyte nuclear factor, *PPAR* = peroxisome proliferator-activated receptor, *SREBP* = sterol regulatory element-binding protein.

^α^*P* = 0.0470 after adjustment (factor 6) *P* = 0.282.

### Association of D19H polymorphism with cholelithiasis

Since individuals with the D19H polymorphism (*rs11887534*, c.52 G > c) in the *ABCG8* gene are known to have a higher risk of developing gallstones [29,41], a genotype analysis of this polymorphism was performed in our cohort. The study population was subdivided into weight- and disease-specific subgroups. Genotype frequencies, odds ratios (ORs) and 95% confidence intervals (CI) for each subgroup were calculated to obtain genotype-associated disease risk estimations (Table 4). Moreover, the genotype-associated disease risk was tested by analysing wild type homozygous (GG) individuals compared to the subjects with the heterozygous type (Gc). Confirming prior reports [29,41], D19H was found to be significantly associated with gallstone disease in our total population with OR = 2.9 (*P* = 0.0220, 95% CI: 1.22–6.89). Notably, overweight simultaneous carriers of gallstones and p.D19H displayed a higher OR (OR = 3.2, *P* = 0.0430, 95% CI: 1.07–9.26) than the respective normal weight group (OR = 1.6, *P* = 0.6270, 95% CI: 0.30–8.77).

**Table 4:** The frequency of the polymorphism D19H in the gallstone carriers and controls of the population from Stuttgart

**Group**	**Controls (134)**			**Gallstone carriers (34)**			**(GG<>Gc)**	
**Genotype**	**G/G**	**G/c**	**c/c**	**G/G**	**G/c**	**c/c**	***P*-value**	**OR (95% CI)**
Total	115	19	0	23	11	0	**0.022**	**2.9**(CI: 1.216 to 6.889)
Normal weight	66	9	0	9	2	0	0.627	1.6(CI: 0.303 to 8.770)
Overweight	49	10	0	14	9	0	**0.043**	**3.2**(CI: 1.071 to 9.264)

*G* = major allele, *c* = minor allele, *OR* = odds ratio, *CI* = confidence interval.

Statistical analysis of genotype frequency differences between gallstone carriers and controls was done using Fisher’s exact test. Odds ratios (ORs) together with a 95% confidence interval (CI) are given as risk measures for the development of gallstones. All statistical tests were two-tailed and a *P-*value of <0.05 was considered as statistically significant.

### Influence of the D19H polymorphism on cholesterol metabolism and intestinal ABCG8 expression

**Figure 2:**
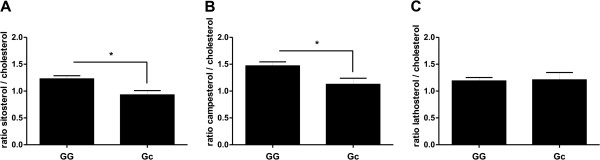
**Influence of the polymorphism D19H on markers of cholesterol metabolism.** Cholesterol absorption is calculated as the ratio of sitosterol or campesterol to cholesterol (**A, B**), cholesterol synthesis is calculated as the ratio of lathosterol to cholesterol (**C**). Values are calculated as means ± SEM. Significance between the wild type individuals and SNP carriers was analysed with Mann–Whitney *U*-test (nonparametric, two-tailed). *P*-values <0.05 were considered as statistically significant. (GG) = wild type individual; (Gc) = carrier of p.D19H; (GG) = 93 and (Gc) = 25. * P_sitosterol_ = 0.0080, *P_campesterol_ = 0.0206.

Next, we analysed the effect of the D19H polymorphism in *ABCG8* on cholesterol absorption and cholesterol synthesis. As shown in Figure 2A and B, carriers of the D19H gallstone risk allele were characterised by significantly reduced intestinal cholesterol absorption of about 24% (sitosterol *P* = 0.0080, campesterol *P* = 0.0206).

**Figure 3:**
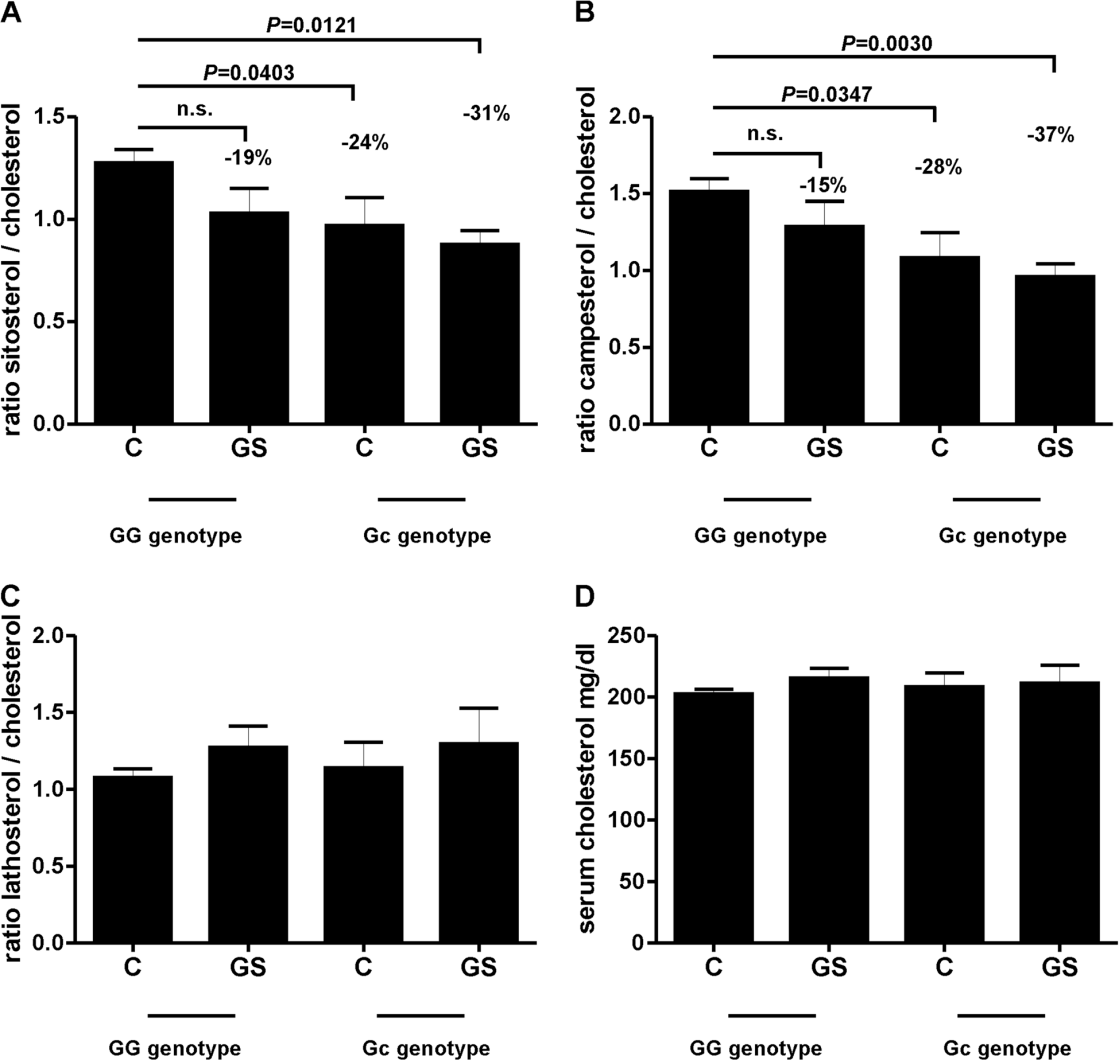
**Influence of the polymorphism D19H on markers of cholesterol metabolism in gallstone disease.** Cholesterol absorption is calculated as the ratio of sitosterol or campesterol to cholesterol (**A, B**), cholesterol synthesis is calculated as the ratio of lathosterol to cholesterol (**C**), total serum cholesterol levels mg/dL (**D**). Values are calculated as means ± SEM. Significance between wild type individuals and SNP carriers was analysed with Mann–Whitney *U*-test (nonparametric, two-tailed). *P*-values <0.05 were considered as statistically significant. C = control subject, GS = gallstone carrier. GG genotype (wild type individual): C = 75, GS = 14; Gc genotype (carrier of p.D19H): C = 18, GS = 11.

After stratifying the Stuttgart population into phenotype- and genotype-specific groups, the influence of the 19H allele on sterol levels was investigated further (Figure 3). The level of cholesterol absorption markers was lower by 15–19% in gallstone carriers with the wild type allele than in the controls (Figure 3A and B). There was no relevant difference in absorption between gallstone carriers and controls with the minor allele. However, independent of gallstone status, cholesterol absorption was consistently lower in carriers of the Gc variant compared with wild type. The most pronounced effect on cholesterol absorption ratio was observed for serum campesterol levels (wild type controls to mutated controls 28%, *P* = 0.0347 and wild type controls to cases with p.D19H 37%, *P* = 0.0030). Regarding the cholesterol synthesis marker lathosterol and serum cholesterol levels, no significant differences were detected between the genotype/phenotype groups (Figure 3C and D).

**Figure 4:**
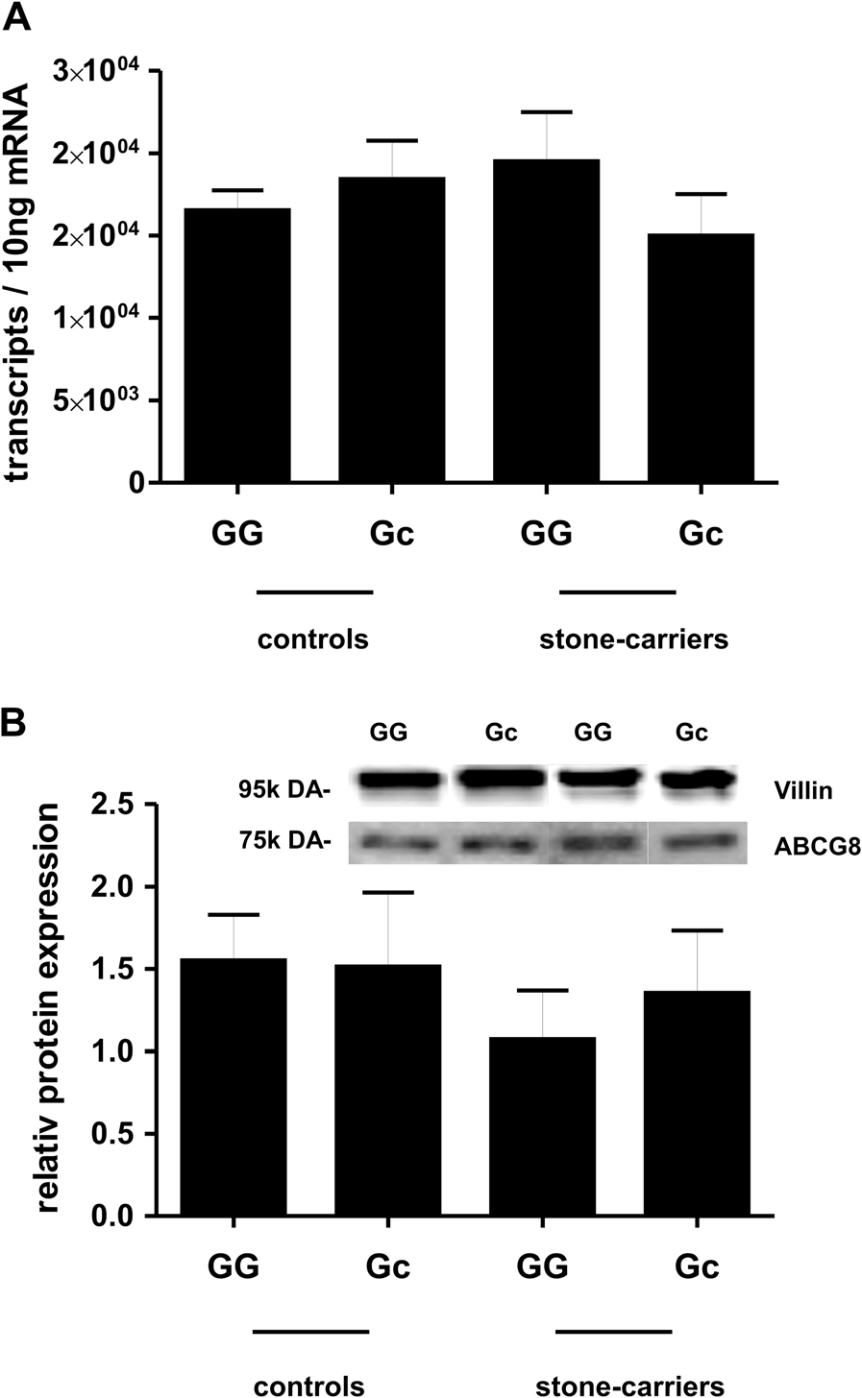
**Correlation of ABCG8 expression to the frequency of the polymorphism D19H in the Stuttgart cohort.** The expression analysis was performed in human ileal mucosal biopsies. Values are given as means ± SEM. Significance between the subgroups was analysed with Mann–Whitney *U*-test (nonparametric, two-tailed). *P*-values <0.05 were considered as statistically significant. (**A**) Quantitative analysis of mRNA expression is calculated as copy numbers. (GG) = wild type individual; (Gc) = carrier of p.D19H. Controls: (GG) = 80 and (Gc) = 15; gallstone carriers: (GG) = 20 and (Gc) = 9. (**B**) Representative Western blot images of ABCG8 protein and villin in ileal mucosa of control individuals and gallstone carriers, with and without p.D19H. Protein content was determined by densitometric analysis. The data were normalized to villin, an epithelial marker protein. Controls: (GG) = 59 and (Gc) = 11; gallstone carriers: (GG) = 11 and (Gc) = 9.

To study whether this polymorphism affected intestinal transporter expression, the intestinal expression of the *ABCG8* gene was related to the D19H subgroups. Ileal ABCG8 levels, mRNA as well as protein features, did not differ between controls with and without the SNP, also not in the gallstone group (Figure 4).

## Discussion

The present study was performed to assess the role of cholesterol metabolism and intestinal transporters as related to the *ABCG8* 19H risk allele in gallstone disease. Four major observations were made: first, cholesterol absorption was decreased in gallstone carriers but cholesterol synthesis was not significantly different between individuals with gallstones and controls. Second, the ileal expression of the sterol transporters ABGG5, ABCG8 and NPC1L1 as well as of their relevant transcription factors is similar between gallstone carriers and controls. Third, in the cohort of gallstone carriers and controls the D19H polymorphism of the *ABCG8* gene was associated with a low cholesterol absorption but not with altered *de novo* synthesis. Fourth, the ileal expression of the *ABCG8* gene is not influenced by the presence of the mutated allele 19H.

Cholesterol and bile acid homeostasis are clearly altered in gallstone patients compared to a healthy population [3]. Most importantly, body weight influences cholesterol absorption and synthesis [42]. Obesity as well as excessive dietary fat and cholesterol uptake are common characteristics in industrial countries and are tightly related to gallstones [43-46]. Although the combination of multiple risk factors and mechanisms is known to contribute to the hypersaturation with cholesterol, the cause for biliary cholesterol hypersecretion is poorly defined. Cholesterol biosynthesis, biliary secretion, intestinal absorption and fecal loss are the major key steps of cholesterol homeostasis but only absorption and *de novo* synthesis determine the net body cholesterol supply. Basically, the absorption efficiency of cholesterol in the small intestine is defined by the concurrent influx and efflux of intraluminal cholesterol molecules crossing the apical membrane [47].

Studies in the murine model with deletion of the *ABCG8* gene observed increased intestinal sterol absorption and reduced hepatic cholesterol secretion [48]. In contrast, overexpression of ABCG5/8 in mice promotes biliary cholesterol secretion and reduces fractional absorption of dietary cholesterol [20]. On the other hand, high cholesterol absorption efficiency and rapid biliary secretion of chylomicron remnant cholesterol enhance cholelithogenesis in gallstone-susceptible mice [49]. Enhanced hepatic expression of ABCG5/8 was already described in Chinese stone-carriers [50] and further studies provide evidence for the functional relevance of hepatic ABCG5/8 expression in relation to the biliary cholesterol secretion [20,51,52].

Measurements of the cholesterol precursor lathosterol allow indirect estimation of *de novo* cholesterol synthesis and the determination of plant sterols (sitosterol and campesterol) reflects intestinal cholesterol uptake [32,33]. Using this technique our findings are clearly consistent with the observation that cholesterol synthesis is generally higher in overweight persons and the efficiency of cholesterol absorption is reduced [43,46]. Moreover, in the current study a diminished cholesterol absorption ratio was noted in gallstone carriers compared to controls. This is in line with previous studies using either the isotope ratio method [3] or also serum surrogate markers [4]. However, there are controversial data regarding cholesterol *de novo* synthesis. The earlier studies found a decreased cholesterol synthesis in gallstone carriers (with high deoxycholate in bile) [11], whereas Kern found an increased synthesis [3]. In the recent study of Krawczyk reduced intestinal absorption concurred with a (possibly compensatory) enhanced hepatic cholesterol *de novo* synthesis [4]. However, in the present study the low intestinal cholesterol absorption was not coupled with enhanced hepatic *de novo* synthesis in gallstone carriers.

Looking for the possible mechanisms determining cholesterol absorption in the gut, the expression of ABCG8, ABCG5 and NPC1L1 as the key intestinal sterol transporters in humans was analysed. Regarding sterol exporter ABCG8 and ABCG5, no significant changes in expression were apparent, also not within the different weight groups. In line with the observation of Masson [53], the levels of ABCG5 correlated only moderately with those of ABCG8 in our study. Both proteins have different distribution profiles along the intestine and can exhibit variable transporter conformations with each other [54]. Recently, a Chinese study demonstrated an increased intestinal expression of NPC1L1 in gallstone patients as a mechanism for upregulated cholesterol absorption in the small intestine [55]. A decreased hepatic NPC1L1 down-regulation in normal weight female gallstone carriers was already described by Cui [56]. The highest expression levels of the transporter were determined in the jejunum [16] which is not easily accessible.

The polymorphism D19H of sterol exporter ABCG8 was associated with gallstones in various populations and different ethnic groups [29,41,57-61]. In line with prior observations the association with gallstones was confirmed in our population. However, overweight carriers of the *ABCG8* 19H risk allele displayed a higher OR than normal weight gallstone carriers. Obesity, of course, doubles the gallstone risk and strengthens the metabolic regulatory differences in cholesterol and bile acid homeostasis [3,9,44,46]. In contrast to the study of Srivastava [59], the D19H relative risk was more pronounced in males than in females (Additional file 1: Table S2). This is probably due to a strong effect from the male overweight gallstone group.

Despite these descriptive studies about the relationship of D19H to the abnormalities in the lipid profile or to gallstones [29,41,62,63], there are no direct cell culture studies on the functional influence of the polymorphism on transporter expression or activity so far. Most prior data, however, are compatible with a gain-of-function hypothesis of the 19H allele contributing to the cholesterol supersaturation of bile and the formation of cholesterol gallstones [27,29]. In our study carriers of p.D19H were characterised by significantly reduced intestinal cholesterol absorption irrespective of the presence or absence of gallstones and the polymorphism did not affect intestinal ABCG8 expression. These results are compatible with the findings of Berge [30] in a normal non-gallstone cohort and those of Gylling showing that hypercholesterolemic carriers of the 19H allele exhibited reduced cholesterol absorption as well [31]. Since ABCG5/G8 are sterol exporters, a gain-of-function would lead to diminished absorption with excessive amounts of cholesterol secreted into intestine as well as bile [29,31,41]. It is unclear why in other cohorts [4] there was no association between the 19H risk allele and intestinal cholesterol absorption and also not with gallstones.

## Conclusions

In summary, both the presence of gallstones and the *ABCG8* polymorphism D19H were related to diminished cholesterol absorption but the polymorphism did not affect ileal expression of ABCG8, suggesting a gain-of-function of the mutated transporter*.* Since cholesterol absorption was also low in gallstone carriers without this polymorphism, this genetic trait does not fully explain this characteristic of gallstone disease.

## Competing interests

The authors declare that they have no competing interests.

## Authors’ contributions

Conceived and designed the experiments: OR SH. Performed the experiments: AS DR DL OR SS. Contributed reagents/materials/analysis tools: OM. Wrote the paper: OR SH. Supervised the study: SH EFS. All authors read and approved the final manuscript.

## Pre-publication history

The pre-publication history for this paper can be accessed here:

http://www.biomedcentral.com/1471-230X/13/30/prepub

## Supplementary Material

Additional file 1: Table S1A Anthropometric and metabolic characteristics of the Stuttgart study cohort, matched cohort (age, gender and BMI). **Table S1B.** Detailed comparison of intestinal cholesterol absorption surrogate markers according to weight. **Table S1C.** Anthropometric and metabolic characteristics of the female Stuttgart study cohort. **Table S1D.** Anthropometric and metabolic characteristics of the male Stuttgart study cohort. **Table S2.** The frequency of the variant D19H in the gallstone carriers and controls of the population from Stuttgart (with subgroups). Click here for file
