# Supplementary material for: Role of the ABCG8 19H risk allele in cholesterol absorption and gallstone disease
Source: BMC Gastroenterol. 2013 Feb 13;13:30. doi: 10.1186/1471-230X-13-30 (PMC3598676; doi:10.1186/1471-230X-13-30)
Supplement: Additional file 1: Table S1A — Anthropometric and metabolic characteristics of the Stuttgart study cohort, matched cohort (age, gender and BMI). Table S1B. Detailed comparison of intestinal cholesterol absorption surrogate markers according to weight. Table S1C. Anthropometric and metabolic characteristics of the female Stuttgart study cohort. Table S1D. Anthropometric and metabolic characteristics of the male Stuttgart study cohort. Table S2. The frequency of the variant D19H in the gallstone carriers and controls of the population from Stuttgart (with subgroups). [file 1471-230X-13-30-S1.doc]

**Supporting information**

**Table 1A Anthropometric and metabolic characteristics of the Stuttgart study cohort, matched cohort (age, gender and BMI)**

|  | **Total 68** | |
| --- | --- | --- |
| **Parameter** | **Controls** | **Gallstone carriers** |
| Number | 34 | 34 |
| Age (years) | 60 ± 1.9 | 61 ± 1.9 |
| BMI (kg/m^2^) | 26.6 ± 0.6 | 26.6 ± 0.6 |
| Total cholesterol (mg/dL) | 217 ± 7.8 | 207 ± 6.2 |
| Total triglycerides (mg/dL) | 147 ± 16.5 | 130 ± 8.4 |
| Lathosterol:Cholesterol (µg/mg) | 1.19 ± 0.12 | 1.33 ± 0.15 |
| Sitosterol:Cholesterol (µg/mg) | 1.23 ± 0.14 | 0.87 ± 0.06↓**^α^** |
| Campesterol:Cholesterol (µg/mg) | 1.52 ± 0.17 | 1.09 ± 0.10↓**^β^** |

BMI=body mass index.

Values are given as means ± SEM (standard error of the mean).

Significance between the controls and gallstone carriers was analysed with Mann-Whitney *U*-test (nonparametric).

*P*-values <0.05 were considered as statistical significant.

**^α^** *P*=0.033 (one-tailed) / reduction of 29%

**^β^** *P*=0.011 (one-tailed) / reduction of 28%

No adjustment and correction in the matched cohort.

**Supporting information**

**Table 1B Detailed comparison of intestinal cholesterol absorption surrogate markers according to weight**

|  | **Normal weight** | | **Overweight** | | **Obese** | |
| --- | --- | --- | --- | --- | --- | --- |
| **Parameter** | **Controls** | **Gallstone carriers** | **Controls** | **Gallstone carriers** | **Controls** | **Gallstone carriers** |
| **Number** | 65 | 8 | 58 | 20 | 11 | 6 |
| **BMI (kg/m^2^)** | 22.3 ± 0.3 | 22.6 ± 0.6 | 27.1 ± 0.2 | 26.5 ± 0.3 | 32.7 ± 1.2 | 32.0 ± 0.9 |
| **Cholesterol absorption (sitosterol)** | 1.38 ± 0.09 | 0.89 ± 0.15**^α^** | 1.11 ± 0.08 | 1.06 ± 0.12 | 0.93 ± 0.15 | 0.85 ± 0.08 |
| **Cholesterol absorption (campesterol)** | 1.68 ± 0.13 | 1.02 ± 0.26**^β^** | 1.32 ± 0.08 | 1.30 ± 0.16 | 1.10 ± 0.17 | 0.96 ± 0.10 |

Normal weight individuals BMI≤24.9, overweight individuals with BMI>25, obese individuals with BMI≥30.

**^α^** *P*=0.0390 after adjustment (factor 6) *P*=0.234

**^β^** *P*=0.0299 after adjustment (factor 6) *P*=0.179

**Supporting information**

**Table 1C Anthropometric and metabolic characteristics of the female Stuttgart study cohort**

|  | **Total** | | **Normal weight** | | **Overweight** | |
| --- | --- | --- | --- | --- | --- | --- |
| **Parameter** | **Controls** | **Gallstone carriers** | **Controls** | **Gallstone carriers** | **Controls** | **Gallstone carriers** |
| **Number** | 61 | 23 | 40 | 8 | 21 | 15 |
| **Age (years)** | **57 ± 1.8** | **64 ± 2.4^α^** | 56 ± 2.3 | 64 ± 4.6 | 58 ± 2.9 | 64 ± 2.8 |
| **BMI (kg/m^2^)** | **24.5 ± 0.6** | **26.7 ± 0.8^β^** | 21.9 ± 0.4 | 22.9 ± 0.7 | 29.4 ± 0.9 | 28.7 ± 0.8 |
| **Total cholesterol (mg/dL)** | 222 ± 6.1 | 205 ± 7.1 | 215 ± 7.1 | 217± 8.2 | **234 ± 10.9** | **198 ± 10^γ^** |
| **Total triglycerides (mg/dL)** | 117 ± 8.6 | 131 ± 10.3 | **110 ± 12.1** | **140 ± 16.7^δ^** | 132 ± 9.8 | 126 ± 13.4 |
| **Lathosterol:Cholesterol (µg/mg)** | 1.13 ± 0.10 | 1.37 ± 0.18 | 1.02 ± 0.13 | 1.27 ± 0.32 | 1.32 ± 0.16 | 1.43 ± 0.23 |
| **Sitosterol:Cholesterol (µg/mg)** | 1.26 ± 0.11 | 0.98 ± 0.11 | 1.37 ± 0.15 | 1.16 ± 0.24 | 1.07 ± 0.16 | 0.87 ± 0.07 |
| **Campesterol:Cholesterol (µg/mg)** | 1.47 ± 0.15 | 1.14 ± 0.14 | 1.63 ± 0.21 | 1.28 ± 0.30 | 1.19 ± 0.15 | 1.04 ± 0.11 |

**^α^***P*=0.0453 (two-tailed) after adjustment (factor 6) *P*=0.272

**^β^***P*=0.0200 (two-tailed) after adjustment (factor 6) *P*=0.120

**^γ^***P*=0.0406 (two-tailed) after adjustment (factor 6) *P*=0.244

**^δ^***P*=0.0435(two-tailed) after adjustment (factor 6) *P*=0.261

**Supporting information**

**Table 1D** **Anthropometric and metabolic characteristics of the male Stuttgart study cohort**

|  | **Total** | | **Normal weight** | | **Overweight** | |
| --- | --- | --- | --- | --- | --- | --- |
| **Parameter** | **Controls** | **Gallstone carriers** | **Controls** | **Gallstone carriers** | **Controls** | **Gallstone carriers** |
| **Number** | 73 | 11 | 35 | 3 | 38 | 8 |
| **Age (years)** | 57 ± 1.3 | 55 ± 2.8 | 55 ± 2.0 | 60 ± 6.1 | 60 ± 1.5 | 53 ± 3.2 |
| **BMI (kg/m^2^)** | 25.9 ± 0.3 | 26.3 ± 0.6 | 23.6 ± 0.3 | 24.2 ± 0.8 | 27.9 ± 0.3 | 27.1 ± 0.5 |
| **Total cholesterol (mg/dL)** | 207 ± 4.5 | 209 ± 12.6 | 206 ± 6.0 | 206 ± 7.1 | 209 ± 6.8 | 211 ± 17.5 |
| **Total triglycerides (mg/dL)** | 135 ± 10.8 | 128 ± 14.9 | 99 ± 6.8 | 85 ± 4.3 | 168 ± 18.33 | 144 ± 17.3 |
| **Lathosterol:Cholesterol (µg/mg)** | 1.16 ± 0.07 | 1.25 ± 0.25 | **0.98 ± 0.08** | 1.03 ± 0.45 | **1.34 ± 0.11^α^** | 1.32 ± 0.31 |
| **Sitosterol:Cholesterol (µg/mg)** | 1.21 ± 0.06 | 0.94 ± 0.10 | **1.36 ± 0.09** | 0.88 ± 0.17 | **1.06 ± 0.08^β^** | 0.96 ± 0.12 |
| **Campesterol:Cholesterol (µg/mg)** | 1.48 ± 0.07 | 1.22 ± 0.19 | **1.70 ± 0.12** | 1.06 ± 0.26 | **1.26 ± 0.09^γ^** | 1.27 ± 0.24 |

**^α^***P*=0.0155 (two-tailed) after adjustment (factor 6) *P*=0.093

**^β^***P*=0.0113 (two-tailed) after adjustment (factor 6) *P*=0.068

**^γ^***P*=0.0069 (two-tailed) after adjustment (factor 6) *P*=0.041

**Supporting information**

**Table 2 The frequency of the variant D19H in the gallstone carriers and controls of the population from Stuttgart (with subgroups)**

| **Group** | **Controls (134)** | | | **Gallstone carriers (34)** | | | **(GG<>Gc)** | |
| --- | --- | --- | --- | --- | --- | --- | --- | --- |
| **Genotype** | **G/G** | **G/c** | **c/c** | **G/G** | **G/c** | **c/c** | ***P*-value** | **OR(95% CI)** |
| **Total** | 115 | 19 | 0 | 23 | 11 | 0 | **0.022** | **2.9(CI: 1.216 to 6.889)** |
| **Females** | 50 | 11 | 0 | 17 | 6 | 0 | 0.543 | 1.6(CI: 0.515 to 5) |
| **Males** | 65 | 8 | 0 | 6 | 5 | 0 | **0.011** | **6.8(CI: 1.677 to 27.338)** |
| **Normal weight** | 66 | 9 | 0 | 16 | 5 | 0 | 0.627 | 1.6(CI: 0.303 to 8.77) |
| **Females** | 34 | 6 | 0 | 6 | 2 | 0 | 0.605 | 1.9(CI: 0.306 to 11.664) |
| **Males** | 32 | 3 | 0 | 3 | 0 | 0 | 1.000 | - |
| **Overweight** | 49 | 10 | 0 | 30 | 15 | 0 | **0.043** | **3.2**(CI: 1.071 to 9.264) |
| **Females** | 16 | 5 | 0 | 11 | 4 | 0 | 1.000 | 1.2(CI: 0.254 to 5.334) |
| **Males** | 33 | 5 | 0 | 3 | 5 | 0 | **0.007** | **11(CI: 1.984 to 60.985)** |

G=major allele, c=minor allele, OR=odds ratio, CI=confidence interval.

Statistical analysis of genotype frequency differences between gallstone carriers and controls was done using Fisher’s exact test. Odds ratios (ORs) together with a 95% confidence interval (CI) are given as risk measures for the development of gallstones. All statistical tests were two-tailed and a *P-*value of <0.05 was considered as statistically significant.
